# Real-Time Monitoring of Water Content in Sandy Soil Using Shear Mode Piezoceramic Transducers and Active Sensing—A Feasibility Study

**DOI:** 10.3390/s17102395

**Published:** 2017-10-20

**Authors:** Qingzhao Kong, Hongli Chen, Yi-lung Mo, Gangbing Song

**Affiliations:** 1 Department of Mechanical Engineering, University of Houston, Houston, TX 77204, USA; qkong@uh.edu; 2 College of Mechanical Engineering and Automation, Zhengjiang SCI-TECH University, Hangzhou 310018, China; chenhldr@zstu.edu.cn; 3 Department of Civil and Environmental Engineering, University of Houston, Houston, TX 77204, USA; ymo@uh.edu

**Keywords:** piezoceramic transducers, active sensing, soil water content monitoring, real time monitoring, wavelet packet analysis

## Abstract

A quantitative understanding of soil water content or soil water status is of great importance to many applications, such as landslide monitoring, rockfill dam health monitoring, precision agriculture, etc. In this paper, a feasibility study was conducted to monitor the soil water content in real time using permanent embedded piezoceramic-based transducers called smart aggregates (SAs). An active sensing approach using a customized swept acoustic wave with a frequency range between 100 Hz and 300 kHz was used to study the wave attenuation in the soil in correlation to soil moisture levels. Two sandy soil specimens, each embedded with a pair of SAs, were made in the laboratory, and the water percentage of the soil specimens was incrementally decreased from 15% to 3% during the tests. Due to the change of the soil water status, the damping property of the soil correspondingly changes. The change of the damping property results in the variation of the acoustic wave attenuation ratios. A wavelet packet-based energy index was adopted to compute the energy of the signal captured by the SA sensor. Experimental results show a parabolic growth curve of the received signal energy vs. the water percentage of the soil. The feasibility, sensitivity, and reliability of the proposed method for in-situ monitoring of soil water status were discussed.

## 1. Introduction

Monitoring of the soil water content in real time is significant for geological and civil applications [1,2]. One example is the landslide, where research has shown that the landslides can be triggered by the increase in the soil moisture caused by intense rainfall or rapid snowmelt [3]. Another example is the soil water monitoring of a rockfill dam, which is essential to ensuring the integrity of the dam [4]. Any excessive seepage without proper action could lead to the failure of the structure. In recent decades, precision agriculture or site-specific crop management in agricultural engineering has become a very important technique, and can increase crop production and quality. In the study of precision agriculture, one of the most attractive research topics is the accurate, on-site and real-time measurement of soil moisture in assuring best crop growth, food production, and disease control [5,6].

A variety of traditional and newly merged technologies have been reported for monitoring the soil water content. The gravimetric soil sampling method is a traditional way to estimate the water content in soil [7]. By measuring the weight difference between the original wet soil and the soil dried by an oven, the water content can be directly calculated. The electrical permittivity of the soil can be used as an index to estimate the soil moisture content [8,9]. By using two or three stainless needles as poles, the local soil electrical permittivity can be measured. Based on the prior calibration of various types of soil specimens, the soil moisture content can be calculated. Many commercialized soil moisture sensors have been designed based on this technology [10]. With the development of electromagnetic technology, researchers began utilizing the electromagnetic waves for soil moisture monitoring [11]. The neutron scatter technique [12,13] and dielectric property measurement [14,15] are commonly used to monitor the near-surface soil moisture in real time. Both approaches have shown the potential to be integrated with Global Positioning Systems (GPS) as satellite remote-sensing technologies to provide real-time global information of soil situation [16]. Though electromagnetic-based satellite remote sensing technologies have been successfully employed for soil moisture monitoring, most of the wave emission equipment is bulky and expensive, which leads to additional labor and economic burden. Acoustic methods can be an alternative solution for soil moisture monitoring with low cost, fast response, and portable equipment. The theories of wave-soil interactions, acoustic wave propagation and attenuation in soil have been investigated [17,18,19]. The relationship between wave velocity and soil moisture has been researched through analytical, experimental, and numerical studies [20,21,22]. This research has shown promising results that the wave speed-moisture curve could be used for determining the soil moisture content. It should be noted that shear wave propagation speed is governed by soil properties, including density, void ratio, effective stress, etc. Related research has been conducted through cross-hole seismic tests [23,24] or bender element tests [25,26]. Due to the great number of soil types, monitoring of the soil water content based on acoustic method requires sufficient calibrations and field tests. In addition, there are two major issues for current acoustic-based soil moisture monitoring. One is the selection of the operating frequencies. In previous research, several of operating frequencies, ranging from several hundred hertz to 30 kHz, have been investigated; however, results have shown that the selected wave propagation was dependent on the wave frequency in soil. Thus, each frequency corresponds to a unique speed-moisture curve. The other issue that limits this technology in real application is the acoustic transducer. To monitor the soil moisture content in practice, the transducer has to be permanently embedded in the soil, which requires the transducer to be robust, reliable, and have certain chemical and environmental resistances.

To address the above two major issues, the authors conducted a feasibility study using an active sensing approach for real-time monitoring of the water content in soil with permanently embeddable piezoceramic-based smart aggregates (SAs) [27]. Previous research has shown the feasibility of using SA to detect the water presence in a concrete crack [28], water seepage in a concrete beam [29], and leakage in a concrete pipe [30]. In this research, a feasibility study was carried out by the authors to study the relationship between the stress wave attenuation and soil water content, so that the results could have the potential to extend the current civil applications of SAs to geotechnical fields, specifically to monitor the soil water content. Instead of several single propagating frequencies, as investigated in previous research, a swept acoustic wave with a frequency range from 100 Hz to 300 kHz was used. Two sand soil specimens were fabricated in the laboratory, and the water content percentage of each was incrementally decreased from 15% to 3% during the tests. The wavelet packet-based energy approach was performed to compute the received signal energy at each water content level. The relationship curves between the wavelet packet-based energy and the water content percentage were obtained. The feasibility, sensitivity, and reliability of the proposed method for in-situ monitoring of the soil water status were discussed. The SA itself used in this research is not original; however, this research is the first to employ SAs and SA-based technologies in soil and to monitor the soil water content in real time, which is considered one of the engineering problems in the geotechnical engineering field.

## 2. Shear Mode Smart Aggregates

**Figure 1:**
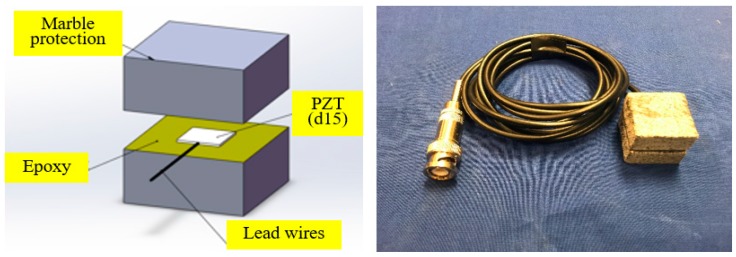
Shear mode SA: (**a**) the structure of a shear mode SA, (**b**) a photo of a shear mode SA.

Due to their capabilities as both actuators and sensors, piezoceramic transducers are versatile, and are commonly used in structural health monitoring. Since the fragile piezoceramic patches can be easily damaged, they cannot be directly deployed as embedded transducers. Song et al. [27] proposed an embeddable piezoceramic-based transducer called smart aggregate (SA), which is designed by casting a waterproofed PZT patch with lead wires by a small concrete cube. Subsequently, Hou et al. [31] used two mating marble blocks to replace the concrete cube to protect the PZT patch. The marble protection further improved the survivability and environmental resistance of the embedded SAs. Previous research has shown that the shear wave is more sensitive in monitoring the materials that subject to water content change [32,33]. Therefore, a shear mode SA, which was designed by sandwiching a shear mode Lead Zirconate Titanate (PZT d_15_) patch between two mating marble blocks, was investigated in this research. Figure 1 gives the structure and a photo of the shear mode SA.

## 3. Principles

### 3.1. Active Sensing Approach 

A stress wave-based active sensing approach using piezoceramic-based transducers has been adopted in structural health monitoring research for many years [27,28,29,30]. In the active sensing approach, selected piezoceramic-based transducers are used as actuators to generate a designed stress wave; meanwhile, others are used as sensors to detect the wave response. The wave response is highly influenced by the geometry or the material property change of the wave medium, so that the received wave signal can be characterized to determine the structural damage or structural condition. In this paper, a shear wave-based active sensing approach using a pair of SAs was investigated to quantify the soil water content. A designed directional shear stress wave with a wide frequency range of 100 Hz to 300 kHz was generated by the SA actuator and the wave response was detected by the SA sensor. Due to the change of the soil water content, the damping property of the soil will change, resulting in the variation of the stress wave attenuation ratios in the soil. The received signal can be captured by the SA sensor and analyzed by using the wavelet packet-based energy approach, which will be introduced below.

### 3.2. Wavelet Packet-Based Energy Approach 

To quantitatively characterize the change of the wave response, a wavelet packet-based energy approach was utilized to compute the signal energy of the received signal [34]. By using the wavelet packet decomposition, the original signal can be decomposed into 2^n^ wavelet packets. Further, the energy of each wave packet can be computed by the sum of the squares of each sampling in the wave packet. Finally, the total energy of the signal can be computed by the sum of all the computed energies of the signal from all the wavelet packets [30]. In this research, the received signal energies corresponding to different water contents in soil were computed using the wavelet packet-based energy approach.

## 4. Experimental Setup

**Figure 2:**
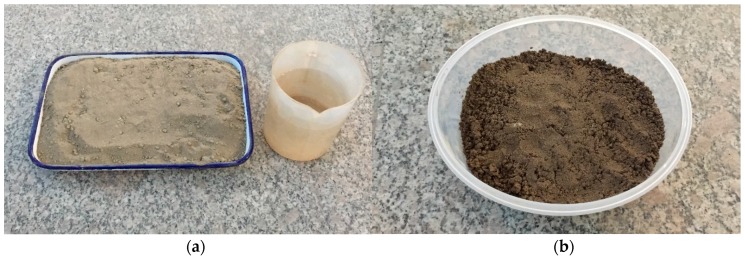
Preparation of the sandy soil. (**a**) Dry sand, (**b**) Sand soil (15% water content).

Figure 2 shows the dry sand (0% water content) and the water-mixed sandy soil. In this research, very fine sand was selected. The particle size was about 0.0625 mm (diameter). A digital weigh scale was used to measure the water content in the sandy soil. The gravimetric moisture content scale is used in this study. The initial water content in the sandy soil was controlled as 15%, which is close to the maximum available water capacity of the sand specimen used in this research [35].

**Figure 3:**
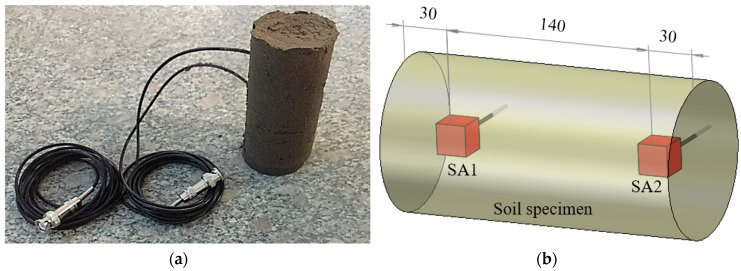
Sandy soil specimen and the location of SAs in the specimen. (**a**) Sandy soil specimen, (**b**) Location of the SAs in the sandy soil specimen.

Two sandy soil specimens were made in the laboratory by using a cylinder mold. Figure 3a shows one sample of the two sandy soil specimens. The diameter and the height of the sandy soil specimen are 100 mm and 200 mm, respectively. In each specimen, a pair of SAs were embedded in the soil. The location of the SAs is shown in Figure 3b.

**Figure 4:**
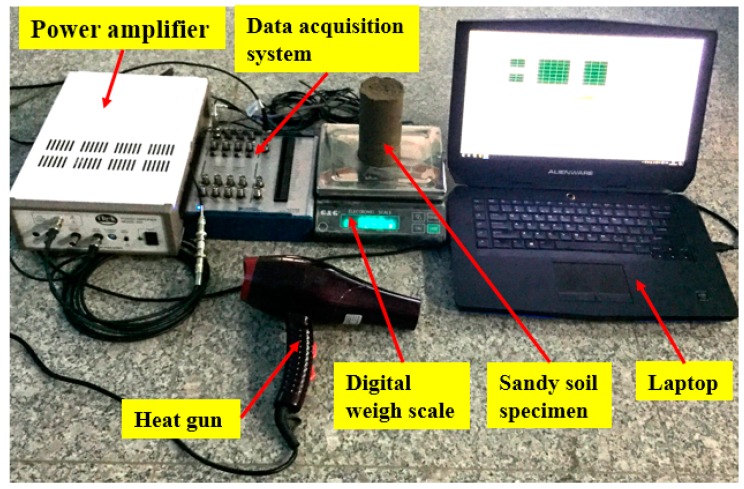
Experimental setup.

The experimental setup is shown in Figure 4. During the test, a heat gun was used to evaporate the water from the sandy soil specimen, so that the water content was reduced. By using the digital weigh scale to measure the weight of the sandy soil specimen in real time, the water content of the sandy soil specimen was incrementally decreased from 15% to 3%. At each integer decrease of the water content, the SA actuator was used to generate the designed shear wave to be received by the SA sensor, and the wave response was collected by the data acquisition system. The frequency range, amplitude, and period of the excitation signal to the SA actuator were 100 Hz to 300 kHz, 3 V, and 1 s, respectively. The gain of the amplifier was 50.

## 5. Experimental Results

### 5.1. Time-Domain Signal Response

**Figure 5:**
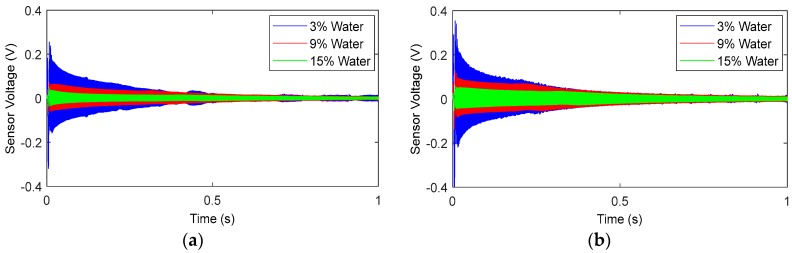
Time-domain signal response of the two test specimens. (**a**) Specimen 1, (**b**) Specimen 2.

The time-domain of the signal responses of the two sandy soil specimens at 15%, 9%, and 3% are shown in Figure 5. It can be seen that, as the water content in the sandy soil specimen decreases, the amplitude of the signal decreases. The possible reason is that, when the water content in the sandy soil specimen decreases, the stiffness of the sandy soil specimen increases, resulting in a decrease in the stress wave attenuation ratio. To quantitatively evaluate the change of the signal response, the wavelet packet-based energy index was computed, as shown in the next section.

### 5.2. Wavelet Packet-Based Energy Index

**Figure 6:**
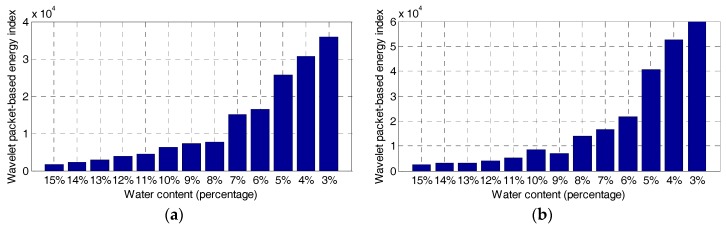
Wavelet packet-based energy index. (**a**) Specimen 1, (**b**) Specimen 2.

The wavelet packet-based energy indices of the two sandy soil specimens are shown in Figure 6. The energy indices obtained from both specimens agree with each other in that, as the water content decreases, the values of the energy indices show the parabolic growth curves. However, at the same water content level of the two specimens, the energy values differ slightly. One possible reason is the inhomogeneity of the two soil specimens, which does have a significant effect on the stress wave propagation between the embedded SAs.

## 6. Discussion

In this research, the feasibility of using SA-based technologies to monitor the soil water content is verified. The combination of the active sensing approach and the durable SAs offers the possibility of employing the proposed approach with embedded sensors to monitor the soil water content in real time. As shown in Figure 6, the energy value of the received signal is parabolically related to the water content in the sandy soil. The two repeated tests yielded similar results. Though the existing electrical permittivity-based technology has been widely applied, the SA-based active sensing approach discussed in this manuscript provides a new concept and solution for this field engineering problem.

It should be also noted that the shear wave propagation characteristics are governed by soil properties, including soil density, void ratio, cementation, size and shape, stress history, density, shapes, void ratios, and other factors [27,28,29]. In addition, the influence of pore-water pressure on the results is not considered in the current study [36]. In this research, the energy values of the two specimens differ slightly at the same water-content level for the two sandy soil specimens. The possible reason for this is the change in the soil-particle arrangement in the two soil specimens. Therefore, significant calibrations are truly required before employing the proposed method for practical applications. Finding an appropriate calibration method to quantify the results with the soil water content remains a challenge for future study. Another point is that the distance change between the SAs do affect the energy of the signal received by the SA sensor. However, the distance change between SAs in this experiment is very limited, such that it would have a neglectable effect on the results. In practical application, two SAs could be attached along a wood/rubber stick to ensure a constant distance. The current research is the conducting of a feasibility study for extending the current applications of piezoceramic SAs to monitoring the soil water content in the geotechnical engineering field. In conclusion, the following research will be studied in the authors’ future work: Firstly, redesign the experimental procedure to ensure a constant water distribution when the soil water content changes. Secondly, investigate more test specimens, including the same soil specimen investigated in the current research, and different soil specimens with more soil types and dimensions. Thirdly, design an appropriate calibration method for giving a quantitative understanding of the soil water content based on the energy value of the SA sensor. In addition, a wider range of water content (3–50%) in soil will be studied to verify the capability and sensitivity of the proposed method in the practical application of soil water content monitoring.

## 7. Conclusions

In this paper, an active sensing approach using shear mode SAs successfully monitored the water content in sandy soil in real time. As the water content decreases in the sandy soil, the amplitude of the corresponding signal increases. The received signal energy values computed by using a wavelet packet-based energy approach is capable of quantitatively monitoring the water content of sandy soil. As shown in the experimental results, the parabolic relationship between the stress wave attenuation and soil water content was found, such that the results could have potentials and provide guidance in extending the current civil applications of SAs in the geotechnical field, specifically for monitoring the soil water content. In the authors’ future work, the sensitivity and reliability of the proposed method will be further investigated. In addition, more soil types and soil conditions will be investigated to verify the validity of the proposed approach. An appropriate calibration method will also be studied.
